# Supplementary material for: Resolving the full spectrum of human genome variation using Linked-Reads
Source: Genome Res. 2019 Apr;29(4):635–45. doi: 10.1101/gr.234443.118 (PMC6442396; doi:10.1101/gr.234443.118)
Supplement: Supplemental Material [file supp_gr.234443.118_Supplemental_longranger-2.2.zip › longranger-2.2.tar.bz2!/lr2.2-public/lib/stan/cmdstan/stan/src/docs/talks/nyc-ml-meetup-2012/img/nuts-ess-2.pdf]

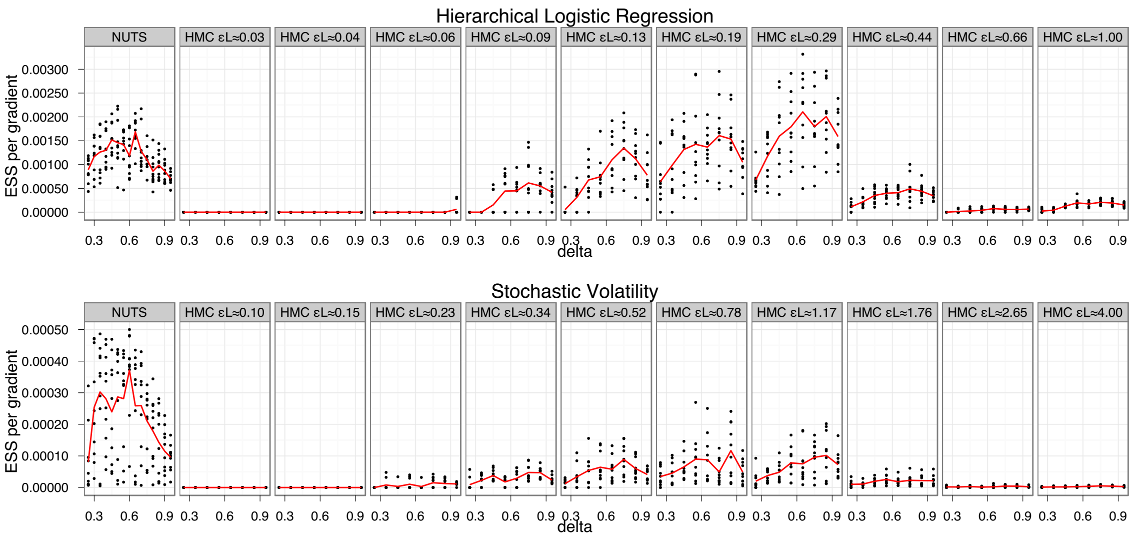

Hierarchical Logistic Regression
NUTS
HMC εL≈0.03
HMC εL≈0.04
HMC εL≈0.06
HMC εL≈0.09
HMC εL≈0.13
HMC εL≈0.19
HMC εL≈0.29
HMC εL≈0.44
HMC εL≈0.66
HMC εL≈1.00
ESS per gradient
0.00300
0.00250
0.00200
0.00150
0.00100
0.00050
0.00000
0.3 0.6 0.9
delta
Stochastic Volatility
NUTS
HMC εL≈0.10
HMC εL≈0.15
HMC εL≈0.23
HMC εL≈0.34
HMC εL≈0.52
HMC εL≈0.78
HMC εL≈1.17
HMC εL≈1.76
HMC εL≈2.65
HMC εL≈4.00
ESS per gradient
0.00050
0.00040
0.00030
0.00020
0.00010
0.00000
0.3 0.6 0.9
delta
